# Molecular genotyping of G6PD mutations for neonates in Ningbo area

**DOI:** 10.1002/jcla.24104

**Published:** 2021-11-11

**Authors:** Jiewen Pan, Danyan Zhuang, Qi Yu, Xiaoli Pan, Youwei Bao, Shuqing Pan, Fei Wang, Lisha Ge, Haibo Li

**Affiliations:** ^1^ Central Laboratory of Birth Defects Prevention and Control Ningbo Women & Children’s Hospital Ningbo China

**Keywords:** cut‐off value, enzyme activity, G6PD, Gene detection, mutation spectrum

## Abstract

The aim of this study is to determine the cut‐off value of glucose‐6‐phosphate dehydrogenase (G6PD) activity and the mutation spectrum of *G6PD* gene in neonates with G6PD deficiency at Ningbo. Around 82233 neonatal blood samples were measured to determine G6PD activity. The positive samples were further detected with gene analysis. A total of 445 neonates were confirmed as G6PD deficiency, and the incidence in Ningbo was 1/185. 17 types of *G6PD* gene mutations were found, including 11 single‐site mutations and 6 double‐site mutations. Considering the significant differences in G6PD activity, the cut‐off value was detected to be 2.35 and 3.65 U/gHb for males and females, respectively. Significant differences in G6PD activities were noted and found to be varied from 4.61 to 6.02 U/gHb in different seasons (*p* < 0.0001). G6PD deficiency screening is a significant detection test for neonatal G6PD deficiency prevention. Our study highlights that the screening should be done using different cut‐off values according to the sexes in different seasons.

## Graphical abstract

Around 82233 neonatal blood samples were measured to determine G6PD activity. The positive samples were further detected with gene analysis. 455 neonates were confirmed as G6PD deficiency, and the incidence in Ningbo was 1/185. 17 types of *G6PD* gene mutations were found, including 11 single‐site mutations and 6 double‐site mutations. Considering the significant differences of G6PD activity, the cutoff value was detected to be 2.35, 3.65 U/gHB for males and females, respectively. Significant differences in G6PD activities were noted and found to be varied from 4.61 to 6.02 U/gHB in different seasons (*p <* 0.0001).

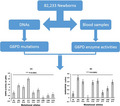

## 1 INTRODUCTION

Glucose‐6‐phosphate dehydrogenase (G6PD) deficiency is an X‐linked (Xq28 chromosome) recessive genetic disorder and considered a hereditary predisposition to hemolysis, which is due to the mutations in the *G6PD* gene (G6PD deficiency is the most common enzyme defect associated in humans with clinical manifestations.^1^ G6PD was discovered in 1956, and to date, G6PD deficiency has influenced around 130–220 million people around the world individually, and more than 200 mutations have been noted.^2^, ^3^ Its mechanism relies mostly on the pentose monophosphate pathway, where G6PD catalyzes the conversion of nicotinamide adenine dinucleotide phosphate (NADP) to its reduced form, NADPH, which further aids in protecting the red blood cells (RBCs) from oxidative damage.^4^ Conventionally, the fluorescent spot test (FST) is widely used for G6PD deficiency screening and its intrinsic disadvantages of plausible positives are widely recognized. G6PD molecular genotypes are believed to be valuable to optimize the cut‐off value of FST screening test in different geographic areas.

The G6PD deficiency varies widely among ethnic groups and it is more prevalent in tropical Africa, Southeast Asia, the Mediterranean, and the Middle East territory, particularly in the Middle East.^5^ In China, the incidence trend of G6PD deficiency is “high in south and low in north,” high prevalence in Guangxi, Guangdong, and Guizhou.^6^, ^7^, ^8^ More than 31 mutation types have been reported in China, and these mutations are 1388G>A, 1376G>T, 95A>G, and so forth, representing 70–80% of the total mutation.^9^ Ningbo City is located in the middle of China's coastline and the climate is similar to the southern wing of the Yangtze River Delta with a moderate latitude, a subtropical monsoon climate, mild and humid. Ningbo has four distinct seasons: 4 months in winter and summer and only about 2 months in spring and autumn. However, the incidence rate of G6PD deficiency in this area is lacking and needs complement to the available data.

In the current study, screening and analyzing genetic variations and mutational frequency of G6PD enzymes in newborns of the Ningbo region was performed and followed by a fluorescent PCR melting curve strategy to affirm whether the identified sample includes a mutation change or not.

## 2 MATERIALS AND METHODS

### 2.1 Experiment objects

A cohort of 82,233 newborns were recruited in 2015 at Ningbo Zhejiang Province. After three days of birth, blood samples were collected for G6PD activity for screening after breastfeeding. The G6PD activity was evaluated every month, and the data were recorded for different seasons. The samples were re‐evaluated with the G6PD activity range of 2.6–3.0 U/gHb, the gene analysis was further subjected for the samples with the G6PD activity lower than 2.6 U/gH.

### 2.2 Detection of G6PD enzyme activity

The enzymatic activity of G6PD was analyzed from the blood samples of newborns. Where the samples were incubated together for 30 mins at room temperature in the presence of Glucose 6 phosphate (G6P) and NADP as described earlier by Stanton.^10^ After adding copper sulfate, the reaction rate was lowered, and the excitation wavelength of 355 nm was set. The fluorescence signal was recorded at an emission wavelength of 460 nm, and the fluorescence intensity of G6PD was measured as described by Stanton.^10^ The G6PD/6PGD ratio was determined as described by Fu et al.

### 2.3 G6PD gene probe detection mutation

The *G6PD* gene mutation is analyzed by detecting the difference in melting temperature between the target DNA molecule and the probe hybridization product. The kit is tested in two tubes for each sample, corresponding to G6PD PCR Mix A and Mix B in the amplification reagents (PerkinElmer Applied Biosystems). The DNA was extracted and subjected to PCR amplification and finally analyzed by melting curve.^11^ To determine the type of mutation, ΔTm value was calculated by finding the difference between normal genes peak versus mutant peak.^11^ The sequence data were compared with a standard reference strain.

### 2.4 Statistical analysis

All the experiments were performed in triplicates, and the results were expressed in mean ±standard deviation (x ± SD). Statistical analysis and Receiver operating characteristics (ROC) curve mapping were performed using GraphPad Prism 7.0. Independent samples that confirmed the normal distribution were analyzed for variance, and a t test was used for comparison between groups. The confidence interval was set to *p* < 0.05.

## 3 RESULTS

A total of 82233 neonates were screened for G6PD deficiency in the Ningbo City of Zhejiang Province. After enzyme activity test, 981 suspected positive neonates (334 males and 647 females) were recalled and further confirmed by gene analysis. Of these, 445 neonates (286 males and 159 females) were confirmed with G6PD deficiency, while the remaining 536 neonates (48 males and 488 females) were negative. The incidence of G6PD deficiency is 1/185, and the ratio for males and females is 1.80:1.

### 3.1 Determination of overall cut‐off value of G6PD

**FIGURE 1:**
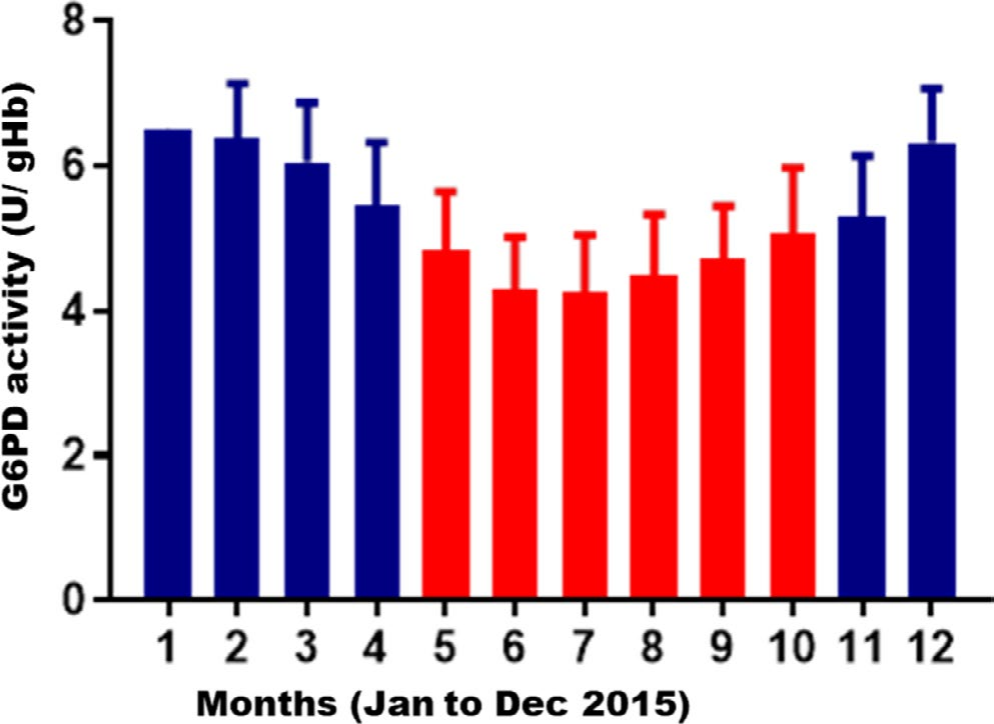
Cut‐off value of G6PD determination

The 445 positive neonates were disease group, and the other 81788 neonates were control group. Further, the ROC curve was established. Figure 1 shows that the area under the curve is 0.9196, the sensitivity and specificity are 90.56% and 93.36% respectively, and the overall cut‐off value is 2.10 U/gHb, which can be considered high sensitivity and specificity.

### 3.2 G6PD activity value in different months

**FIGURE 2:**
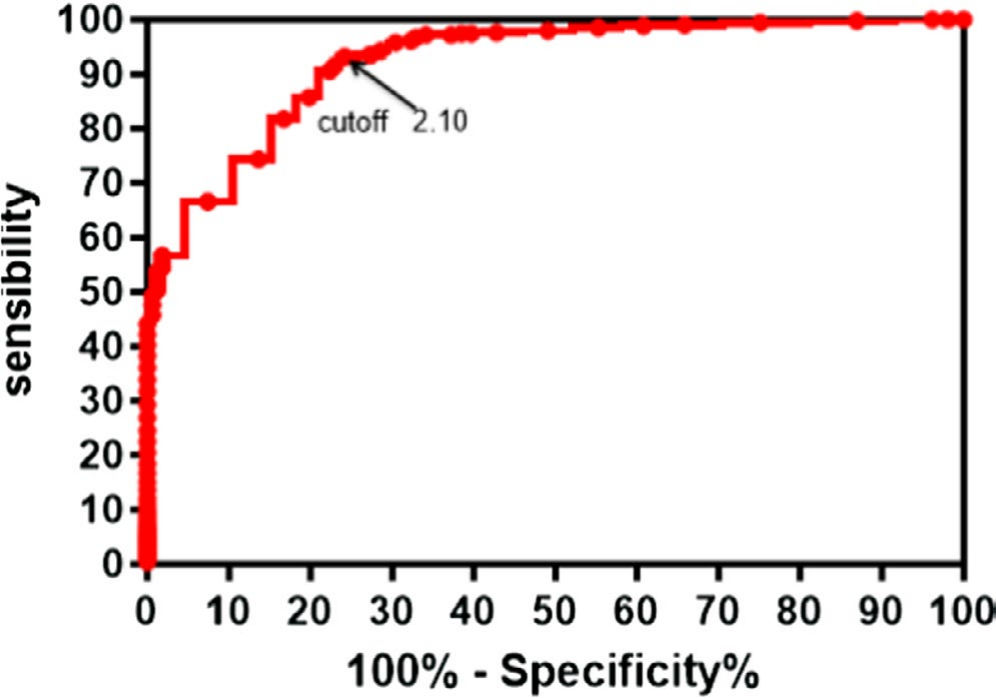
G6PD activity during different seasons of a year (2015) at Ningbo city of Zhejiang

The experiment of G6PD activity was performed every month, and data were analyzed (Figure 2). The G6PD enzyme activity value was varied among seasons, the mean value of enzyme activity is about 4.61 U/gHb during the summer and autumn (May‐October) and 6.02 U/gHb during spring and winter (January‐April, November‐December) (*p *< 0.0001). Seasonally, the infants born in summer and autumn had lower G6PD level than those who were born in spring and winter.

### 3.3 G6PD critical values for different sexes

**FIGURE 3:**
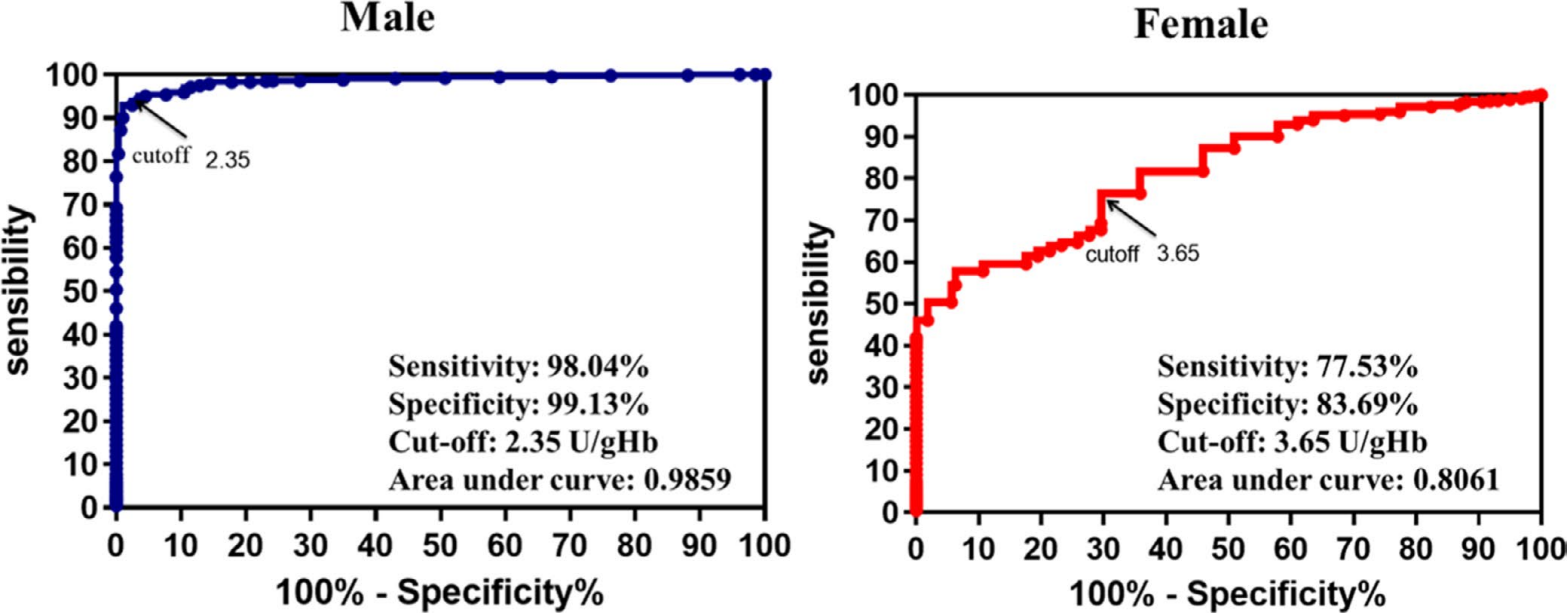
Determination of cut‐off values for G6PD activity through ROC curve analysis in both male and females

The G6PD enzyme activity value was varied between males and females (*p *< 0.0001) among the analyzed samples. In this study, 286 positive males were taken as the disease group, 48 negative males were negative group and the neonates with normal initial screening value were the control group. In the analyzed female samples, 159 neonates were diagnosed and were disease group. Further, the 488 negative female cases were negative group and the neonates with normal initial screening value were the control group. The ROC curve was established. A cut‐off value of 2.35 U/gHb in men and the area under the curve was 0.9859, the sensitivity and specificity were 98.04% and 99.13%, the cut‐off value for females was 3.65 U/gHb, the area under the ROC curve was 0.8061, and the sensitivity and specificity were 77.53% and 83.69% (Figure 3).

### 3.4 Mutational analysis of *G6PD* gene

**FIGURE 4:**
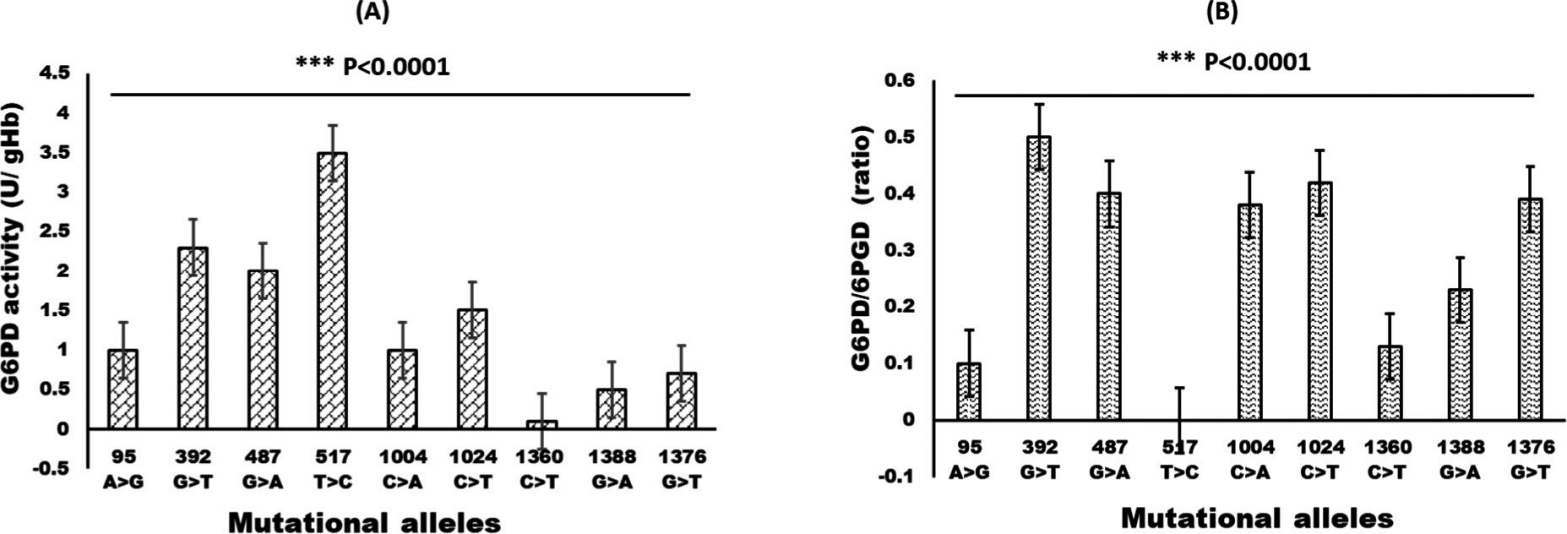
Correlational analysis of G6PD activity (A) and G6PD ratio among G6PD mutational alleles. The data obtained had shown be statistically different for enzyme activity and G6PD ratio (*p *< 0.0001)

In this study, 17 different types of mutations were detected, including 11 single‐site mutations and six double‐site mutations (Table 1). Statistical significance was found when compared with the negative control group (*p *< 0.05). Where, c. 1376G>T complex c. 1024C>T; c. 1376G>T complex c. 392G>T; c. 487G>A complex c. 392G>T; c. 871G>A complex c. 1024C>T; c. 95A>G complex c. 1024C>T mutation 1 case each, accounting for 0.22%; c. 95A>G complex c. 1388G>A mutation 2 cases, accounting for 0.44% (Table 1). Nine mutations were considered for correlational analysis for both enzyme activity and G6PD ratio as shown in Figure 4. It was determined that the mutational allele, ie, c.517 T>C enzyme activity with higher activity, had a negligible ratio. However, various alleles with mutations had various enzyme activity.

**TABLE 1:** Distribution of mutational alleles among 445 neonates with G6PD mutations

	Cases noticed	Proportion of various variants (%)
Variant alleles		
c.95A>G	56	12.58
c.392G>T	17	3.82
c.487G>A	3	0.67
c.517T>C	3	0.67
c.592C>T	3	0.67
c.871G>A	30	6.74
c.1004C>A	6	1.35
c.1024C>T	71	15.95
c.1360C>T	3	0.67
c.1388G>A	115	25.84
c.1376G>T	131	29.44
c.383T>C	0	0
Complex variant alleles		
c.1376G>T complex c.1024C>T	1	0.22
c.1376G>T complex c.392G>T	1	0.22
c.487G>A complex c.392G>T	1	0.22
c.871G>A complex c.1024C>T	1	0.22
c.95A>G complex c.1024C>T	1	0.22
c.95A>G complex c.1388G>A	2	0.44
Total	445	

## 4 DISCUSSION

The global incidence of G6PD deficiency is around 4.95% and was distributed unevenly. The incidence of this disease in America comes to 3–4%, Europe 3–9%, the Pacific Ocean 2–9%, South of Sahara (an African country) 5–7%, Middle East 0–6%, and Asia 4–7%. In China, the incidence of disease in Chinese Taipei has reached to 3.1–9.0% and 4.4% in Hong Kong territory.^5^ As for mainland regions of China, G6PD deficiency can be found in the south of the Yangtze River, featuring a higher incidence of rate in the south and lower one in the north.^5^, ^12^ The southern regions of China including Guangdong, Guangxi, Guizhou, Hainan, and Yunnan are highly incidence provinces.^5^ The occurrence of disease was found to be quite low from the northern area of Chongqing and Wuhan.^13^ The places like Shandong and Liaoning had very few incidences of G6PD deficiency. Nevertheless, with the advancement of current transportation, the mass movement of populace starting with one region then onto the next over China, lead to an increase in G6PD deficiency prevalence. For example, there is an increase of patient suffering from G6PD in the northern area of China, while the cases in new infants have reached 0.2% in Shanghai. In this study, the distribution and phenotypes of mutations causing G6PD deficiency in Ningbo area is firstly investigated. 82,233 neonates were screened for G6PD deficiency in the Ningbo City of Zhejiang Province. 445 neonates were diagnosed with G6PD deficiency, including 286 males and 159 females, and the incidence in Ningbo was 1/185. The incidence of G6PD deficiency is close to Shanghai (0.2%). Chen et al.^13^ have suggested that G6PD deficiency was more prevalent in male patients in China. The results of our study 184 are consistent with the report.

Although most patients with G6PD deficiency do not have any symptoms, the intake of food and drugs will cause serious hemolytic reaction and endanger the life of the newborn. Early diagnosis and prevention must be made against G6PD deficiency. In China, newborn screening of G6PD deficiency is carried out by measuring G6PD enzyme activity. When the value is lower than the cutoff, it is classified as a plausible positive, where a further validation such as G6PD molecular genotyping will be required. A reliable and area‐specific screening cutoff becomes significant in newborn screening of G6PD deficiency. The results of our study showed that the activity of G6PD was affected by season and sex. According to the G6PD cut‐off value from ROC curve in this research, the male neonate's cut‐off value was 2.35 U/gHb. The size under ROC curve is 0.9859, which indicates the cut‐off value recognizes the majority of patients. The sensitivity and specificity amount to 98.04% and 99.13% respectively. In terms of female, the female neonate's cut‐off value was 3.65 U/gHb. The size under ROC curve is 0.8061. The sensitivity and specificity amount to 77.53% and 83.69% respectively. When we use 2.35 U/gHb as critical value for male infants, we find that its sensitivity is higher than 2.10 U/gHb. The result also shows that the sensitivity of 3.65 U/gHb is higher than 2.10 U/gHb when using high cut‐off value for female infants. In reference to our result and the others, it is advisable that critical value in G6PD for female be increased to 3.65 U/gHb, which can recognize additional G6PD patients. And for male, 2.35 U/gHb will be used for cut‐off value. In addition, the G6PD enzyme activity varies among different seasons. During hot summer and autumn season, the critical value should be set lower and the figure reaches 2.10 U/gHb. In cold spring and winter season, the critical value should be set higher, which reaches 2.35 U/gHb. Overall, the screening should be done specifically for different sexes in different seasons. For instance, the cut‐off value for males should be lower than females in the same season; as for female samples, it should be adjusted accordingly for different reasons. G6PD genotyping shall be utilized to compliment the conventional FST to improve the clinical diagnosis and verification.

According to the report of gene tank, more than 200 types of mutation have been reported, and 90% are single‐site mutation. About 30 types of mutation are found in Asian.^14^ 981 suspected positive neonates were recalled for gene analysis. 445 neonates were diagnosed with G6PD deficiency, and 17 gene types were detected. The c.1376G>T, c.1388G>A, c.1024C>T, and c.95A>G are common gene mutation. This mutations accounts for 83.81% of total gene mutation. According to reports, c.95A>G, c.1376G>T, and c.1388G>A are most common three mutation types in our country, and they account for 60–72% of domestic G6PD mutation. In this research, it is found that c.1376G>T is the main mutation type in the city of Ningbo. c.1388G>A and c.1024C>T comes as the second and third. The result of our study is close to the reports, but mutation type such as c.1024C>T occurs on a more frequent basis, which comes as the third place. Subsequently, we analyze parents’ place of origin who carry mutation and it is found that mothers are mostly from Guizhou Province. Wu had reported that c.1024C>T is one of the most frequent mutations in Guizhou population.^11^ China covers a vast area with many nationalities. There is a discrepancy in terms of G6PD mutation in different areas and different regions. And in this research, we even find that those less common mutation types such as 1360C>T、487G>A、517T>C, which is an indication of various mutation and different types in Ningbo area, which also has its own regional feature. At the same time, six double‐sites mutations are found, but the frequency of occurrence is quite rare. A study conducted by Chen et al.^13^ among 1813 Chinese samples screened, 15 single mutations, and 7 double‐sites mutations were noted with no frameshift. However, in our study, 17 different *G6PD* gene mutation types were observed, and the enzyme activity in different individuals has not affected by the same kind of *G6PD* gene mutation. Further, our analysis on the G6PD/6PGD ratio was in accordance with Fu et al., where our mutational alleles in the *G6PD* gene and enzyme activity were statistically different (*p *< 0.0001). The current study suggested that there is no disparity between phenotype and genotype. Though, in a few cases, the difference between genotype and phenotype was noticed among Chinese G6PD deficient patients.^13^

## 5 CONCLUSION

In our investigation, a large cohort of samples were screened from the Ningbo area for G6PD deficiency for the first time. The mutational analysis of G6PD combined with the screening method of bio‐chemistry testing was found to improve the effect of hemizygote testing. Our study has further highlighted the screening should be done specifically for different sexes in different seasons.

## Data Availability

The data that support the findings of this study are available from the corresponding author upon reasonable request.
